# Prevalence of generalized joint hypermobility, musculoskeletal injuries, and chronic musculoskeletal pain among American university students

**DOI:** 10.7717/peerj.7625

**Published:** 2019-09-11

**Authors:** Peter R. Reuter, Kaylee R. Fichthorn

**Affiliations:** Department of Rehabilitation Sciences, Marieb College of Health & Human Services, Florida Gulf Coast University, Fort Myers, FL, USA

**Keywords:** Chronic musculoskeletal pain, Beighton score, Musculoskeletal injuries, Generalized joint hypermobility

## Abstract

The objective of this study was to investigate the prevalence of generalized joint hypermobility (GJH) in a university-aged population, whether young adults (aged 18–25 years) with GJH are prone to sustain more musculoskeletal injuries, and are more likely to suffer from chronic musculoskeletal pain. The study used an interactive survey to gather data; GJH was assessed using a cut-off Beighton score of ≥5 in accordance with the 2017 International Classification of EDS criteria. The analyzed sample consisted of 482 female and 172 male participants from Florida Gulf Coast University (USA). The prevalence of GJH in a university-aged population can be estimated at 12.5%. Women did not have higher rates of GJH than men. However, female participants showed significantly higher rates of hypermobility of the spine as well as the right knee and elbow joints. The Beighton scores did not differ by ethnicity/race. Female participants had a lower rate of self-reported injuries than male participants, although this difference was not significant. There was no difference in the proportion of all participants classified within different categories (0; 1–4; 5–9) of Beighton scores and whether or not they reported having been injured. Male and female participants reported chronic pain of joints and neck or back at the same rates across the Beighton score categories. Female participants, however, reported higher pain intensity for chronic neck and back pain. This study increases knowledge about a correlation between GJH, musculoskeletal injuries, and chronic pain of joints, neck, and back in a university-aged population.

## Introduction

Although joint laxity or hypermobility is a well-known condition, there has long been a lack of a universally acknowledged definition or terminology (Remvig et al., 2014). An excessive range of motion across multiple joints was usually referred to as generalized joint hypermobility (GJH) or generalized joint laxity (GJL). The terms joint hypermobility syndrome, benign joint hypermobility syndrome (BJHS), and hypermobility syndrome were used to describe a disorder characterized by musculoskeletal symptoms, such as chronic joint or ligament pain or osteoarthritis, due to joint hypermobility (Kumar & Lenert, 2017; Morris et al., 2017).

Beighton & Horan (1969) proposed a scoring system for joint hypermobility, which was a revision of the system proposed by Carter & Wilkinson (1964). The scoring system currently used in most epidemiologic studies of GJH was described by Beighton, Solomon & Soskolne (1973). It consists of a series of nine dichotomous joint extensibility tests (Table 1), where a tested joint is either hypermobile (score = 1) or not hypermobile (score = 0). Therefore, the total score (Beighton score) lies between 0 and 9, with higher scores indicating greater joint laxity. The test has a moderate to high inter-tester repeatability (Junge et al., 2013) and demonstrated validity and reliability in a number of studies (Morris et al., 2017; Bulbena et al., 1992; Smits-Engelsman, Klerks & Kirby, 2011; Juul-Kristensen et al., 2007).

**Table 1:** The Beighton criteria for joint hypermobility.

Passive dorsiflexion of the little fingers beyond 90° (one point for each hand)—two points Passive apposition of the thumbs to the flexor aspects of the forearm (one point for each thumb)—two points Hyperextension of the elbows beyond 10° (one point for each elbow)—two points Hyperextension of the knee beyond 10° (one point for each knee)—two points Forward flexion of the trunk with knees fully extended so that the palms of the hands rest flat on the floor—one point

In the past, a Beighton score of ≥4 was often used to indicate GJH in adults, although there were studies that used cut-off scores of ≥3, ≥5 or ≥6 (Morris et al., 2017; Beighton, Solomon & Soskolne, 1973; Bulbena et al., 1992; Russek, 1999). Because joint laxity is greatest in infants and then gradually decreases during childhood and adolescence, a higher threshold was advocated for use in children (Morris et al., 2017; Smits-Engelsman, Klerks & Kirby, 2011; Middleditch, 2003; Clinch et al., 2011; Jansson et al., 2004; Van Der Giessen et al., 2001; Malfait et al., 2017; Scheper et al., 2014; Remvig et al., 2011; Remvig, Jensen & Ward, 2007). In 2017, the International Consortium on the Ehlers-Danlos syndromes (EDSs) proposed to use the following cut-off Beighton scores for the diagnosis of GJH: ≥6 for pre-pubertal children and adolescents, ≥5 for pubertal men and women up to the age of 50, and ≥4 for those >50 years of age (Malfait et al., 2017).

Symptomatic as well as asymptomatic joint hypermobility is due to inherited alterations of proteins that lead to a laxity of connective tissue, although there is no consensus on the underlying pathophysiology (Scheper et al., 2014; Remvig et al., 2011; Remvig, Jensen & Ward, 2007). Heritable disorders of connective tissue (HDCTs) are caused by mutations in genes that code for proteins of the connective tissue matrix, such as collagens, fibrillins, elastins, and proteoglycans (Baeza-Velasco et al., 2011; Collinge & Simmonds, 2009; Grahame, 2000a; Malfait et al., 2006). These changes to the connective tissue matrix affect the stability of joint capsules and the extensibility of ligaments and tendons. Classic HDCTs, such EDS, Marfan syndrome, and osteogenesis imperfecta, may lead to serious clinical symptoms and even cause premature death in affected individuals (Scheper et al., 2014; Remvig et al., 2011). The EDS classification proposed by the International Consortium on the EDSs lists GJH as a one of two major criteria for classical EDS, with skin hyperextensibility and atrophic scarring being the other major criterion. GJH is also listed as a major criterion for classical-like EDS and hypermobile EDS (hEDS) (Malfait et al., 2017). The International Consortium on EDS also suggested use of the term hypermobility spectrum disorder (HSD) for individuals with GJH who do not suffer from EDS or hEDS (Malfait et al., 2017; Castori et al., 2017). GJH without musculoskeletal symptoms is considered asymptomatic GJH; GJH with musculoskeletal symptoms, generalized HSD (Castori et al., 2017). If only a small number of joints is hypermobile, i.e., the Beighton score is <5 for adult men and women up to the age of 50, the condition may be called localized joint hypermobility (LJH). LJH usually affects one or two smaller or larger joints only, and may be bilateral, such as in bilateral genu recurvatum. In peripheral joint hypermobility (PJH), the hypermobility is typically limited to the hands and/or feet. Unlike GJH, which is most often congenital and based on an inherited trait, localized and PJH can be acquired conditions, such as spine hypermobility in gymnasts and other athletes (Castori et al., 2017).

As the underlying pathophysiology affects not only the musculoskeletal system but also other body organs and systems, such as the cardiovascular system, the so-called Brighton criteria for the diagnosis of BJHS, that combine Beighton scores and clinical scores, were proposed in 1998 (Grahame, Bird & Child, 2000). A Beighton score of ≥4 was a major criterion of the Brighton criteria. The criteria were used to assess the prevalence of BJHS. Both the Brighton criteria and the term BJHS are no longer in use because of the new EDS classification.

The prevalence of GJH in child and adult populations has been reported to range from 2% to almost 65% (Scheper et al., 2014; Remvig et al., 2011; Rikken-Bultman, Wellink & Van Dongen, 1997; Simmonds & Keer, 2007; Lamari, Chueire & Cordeiro, 2005; Leone et al., 2009). The divergence of these results is mainly due to the various methods of evaluation (Beighton score, Brighton criteria) and different cut-off Beighton scores used (see above), the different populations studied as well as the fact that joint laxity is highest during early childhood and continues to decrease during adolescence and adult life (Middleditch, 2003). Therefore, the younger the studied population, the higher the reported prevalence of GJH (Beighton, Solomon & Soskolne, 1973; Rikken-Bultman, Wellink & Van Dongen, 1997; Arroyo, Brewer & Giannini, 1988; Juul-Kristensen et al., 2009; Alejo et al., 2009; El-Garf, Mahmoud & Mahgoub, 1998; Hasija, Khubchandani & Shenoi, 2008; Seçkin et al., 2004). Studies that used a cut-off Beighton score of ≥4 report prevalence rates of approximately 35% for populations consisting of children and adolescents (3–18 years of age) (Smits-Engelsman, Klerks & Kirby, 2011; Arroyo, Brewer & Giannini, 1988; Juul-Kristensen et al., 2009; Qvindesland & Jonsson, 1999). The prevalence of GJH in the adult population has been reported to range from 10% to 30% (Scheper et al., 2014; Collinge & Simmonds, 2009). Baeza-Velasco et al. (2011) found a prevalence of 39.5% of BJHS among French university students using the Brighton criteria. In Chile, the prevalence was found to be 39%, while GJH is reported to constitute approximately 25% of rheumatologic cases (Baeza-Velasco et al., 2011; Bravo & Wolff, 2006; Gumà et al., 2001). Russek & Errico (2016) reported an overall prevalence of 26.2% for GJH in a healthy university student population using a cut-off Beighton score of ≥5 and a prevalence of 19.5% for BJHS using the Brighton criteria.

Regardless of the criteria used and the age of the population studied, the prevalence of GJH and BHJS among females is higher than in males (Beighton, Solomon & Soskolne, 1973; Jansson et al., 2004; Grahame, Bird & Child, 2000; Arroyo, Brewer & Giannini, 1988; Russek & Errico, 2016; Wordsworth et al., 1987; Jessee, Owen & Sagar, 1980; Al-Rawi, Al-Aszawi & Al-Chalabi, 1985; Larsson, Baum & Mudholkar, 1987; Gedalia et al., 1985; Decoster et al., 1997; Hakim, Malfait & Paepe, 2010). However, this does not apply to all joints used as part of the Beighton score (Cameron et al., 2010; Didia, Dapper & Boboye, 2002). Generalized hypermobility was also reported to be higher among Africans than people of European descent and to be higher among Asians than Africans (Russek, 1999; Wordsworth et al., 1987; Hakim, Malfait & Paepe, 2010; Grahame, 1990).

Many individuals with GJH remain asymptomatic throughout their lives (Kirk, Ansell & Bywaters, 1967). Sometimes they even take advantage of their hypermobility to excel in sports such as ballet (Grahame & Jenkins, 1972) or dancing (Day, Koutedakis & Wyon, 2011; Scheper et al., 2013). But, they may also be at an increased risk for musculoskeletal injuries, for example, sports-related injuries to the ankle, knee and shoulder joints (Cameron et al., 2010; Scheper et al., 2013; Decoster et al., 1999; Smith et al., 2005; Beynnon, Murphy & Alosa, 2002; Borsa, Sauers & Herling, 2000; Wolf, Cameron & Owens, 2011; Konopinski, Jones & Johnson, 2012; Pacey et al., 2010). Compared to individuals without joint hypermobility, adult individuals with GJH are reported to have a higher rate of osteoarthritis of hip, knee, and hand joints (Konopinski, Jones & Johnson, 2012). Individuals with GJH have an increased risk of injuries to the anterior cruciate ligament (ACL) (Ramesh et al., 2005). Female athletes with GJH have a five times greater risk for knee injuries than female athletes without GJH (Myer et al., 2008). While it has been shown in some studies that GJH is associated with more musculoskeletal injuries, there are, however, studies that show a similar or reduced injury risk for athletes with GJH (Scheper et al., 2013; Decoster et al., 1999; Nicholas, 1970; Stewart & Burden, 2004; Krivickas & Feinberg, 1996).

The main complaint of individuals with symptomatic GJH is chronic musculoskeletal pain (MSP) that may affect their daily activities, leading to a decreased quality of life (Kumar & Lenert, 2017; Morris et al., 2017; Leone et al., 2009; Hakim, Malfait & Paepe, 2010; Scheper et al., 2013; Chustecka, 2004; Grahame, 2000b; Simonsen et al., 2012; Nikolajsen et al., 2013). Some individuals complain of pain in several joints (Grahame & Jenkins, 1972), fatigue (Voermans et al., 2010), muscle weakness (Scheper et al., 2014; Engelbert et al., 2003), or diminished motor performance (Hanewinkel-Van Kleef et al., 2009). Yet, a systematic review of GJH and MSP in children did not show an association in Caucasian populations and only a potential association in African and Asian populations (McCluskey et al., 2012). While some longitudinal studies seem to support an association between GJH and MSP, they have not provided evidence for GJH being a causative factor for MSP (El-Metwally et al., 2004; Tobias et al., 2013; Sohrbeck-Nohr et al., 2014).

Since the Beighton criteria were introduced more than 45 years ago, only a few studies have been published that looked at the overall prevalence of GJH among child or adult populations in North America. Jessee, Owen & Sagar (1980) published a study in 1980 showing a prevalence of hypermobility of 4.9% among 637 blood donors. They also reported that there were no statistically significant differences for arthritis/arthralgia and joint complaints between participants with and without hypermobile joints. A study involving students of the freshman class of the United States Military Academy at West Point, NY found a prevalence of 1.5% for GJH using a cut-off score of ≥4 (Cameron et al., 2010). Russek & Errico (2016) reported an overall prevalence of 26.2% for GJH using a cut-off Beighton score of ≥5 in 267 undergraduate and graduate students aged 17–26. The study also found that GJH was not associated with an increased incidence of musculoskeletal injuries. Other studies published within the last two decades looked at specific groups, such as NCAA lacrosse players (Decoster et al., 1999), junior netball players (Smith et al., 2005), and female soccer players (Blokland et al., 2018).

This study used data gathered from a convenience sample of 686 male and female students at an American university to determine the prevalence of GJH, injuries to the musculoskeletal system, and of chronic MSP in this population. The study aimed to answer the question whether young adults with GJL are prone to sustain more injuries to joints, ligaments, tendons, and muscles, and are more likely to suffer from chronic MSP.

## Methods

### Ethical research statement

The research protocol and its amendment were approved by an ethical review board (Institutional Review Board; IRB) at Florida Gulf Coast University prior to data collection (FGCU; IRB 2014-64, November 17, 2014; IRB 2014-64 amendment, November 9, 2015). All researchers involved in data collection were trained in ethical data collection through the Collaborative Institutional Training Initiative. Data collection followed all laws relevant to the survey of university student populations.

### Data collection

Data were collected between January 2016 and October 2017 using an interactive survey administered to undergraduate students enrolled in Human Anatomy & Physiology with lab I classes at FGCU in Fort Myers, Florida (USA). Students in the course were asked to participate in an anonymous survey during the lab session on Skeletal System/Joints of the Spring 2016, Fall 2016, Spring 2017, and Fall 2017 semesters. Students enrolled in the course are usually pre-health professions students, who have to complete the course successfully before applying for admission to restricted programs, such as Nursing or Athletic Training, or to stay in their current major (e.g., Exercise Science and Health Sciences). The students first studied the general structure of joints, joint physiology, and range of motion (including joint hypermobility), before completing the survey. They assessed each other’s joints as a group assessment (two to four students working together) using goniometers under supervision by trained members of the research team.

The cover page of the survey consisted of an IRB approved consent form; in other words, written consent was obtained. Participation in the study was completely voluntary and students were free to change their mind and stop participation at any time, for any reason, without penalty or loss of any future services they may be eligible to receive from the FGCU. Approximately 1,350 surveys were handed out to students, 686 surveys were deposited anonymously in the survey collection box and included in the study. The complete survey can be found in Appendix S1.

The survey consisted of five groups of questions. The first group of questions collected information about the participants’ hand grip strength of the right and left hand as well as which of their joints (spine, knees, little fingers, thumbs, elbows) were hyperextensible. The study used a digital electronic dynamometer to measure hand grip strength. Participants were asked to measure the grip strength of each hand twice. They worked in groups of two to four students to evaluate each other’s joints for hypermobility under supervision by members of the research team.

The second group of questions asked for demographic information, such as gender, age, ethnicity/race, handedness, and footedness. The third group asked questions about athletic involvement, and the fourth group about the history of joint, ligament/tendon, and muscle injuries of the participants as well as past or current hernias and herniated discs in their back or spine. The last group of questions focused on the medical history of the participants, including chronic MSP, as well as their family medical history in regard to medical conditions that could be related to benign hypermobile joint syndrome and underlying conditions, such as connective tissue disorders.

Information provided on musculoskeletal injuries was used to group the injuries using the International Classification of Diseases, Tenth Revision, Clinical Modification (ICD-10-CM; Table 2) (Centers for Disease Control and Prevention, 2018).

**Table 2:** ICD-10-CM codes used to group self-reported injuries.

ICD-10-CM code	Description
S03	Dislocation and sprain of joints and ligaments of head
S16	Injury of muscle, fascia, and tendon at neck level
S29	Other and unspecified injuries of thorax
S39	Other and unspecified injuries of abdomen, lower back, pelvis, and external genitals
S42	Fracture of shoulder and upper arm
S43	Dislocation and sprain of joints and ligaments of shoulder girdle
S46	Injury of muscle, fascia, and tendon at shoulder and upper arm level
S52	Fracture of forearm
S53	Dislocation and sprain of joints and ligaments of elbow
S56	Injury of muscle, fascia, and tendon at forearm level
S62	Fracture at wrist and hand level
S63	Dislocation and sprain of joints and ligaments at wrist and hand level
S73	Dislocation and sprain of joint and ligaments of hip
S76	Injury of muscle, fascia, and tendon at hip and thigh level
S82	Fracture of lower leg, including ankle
S83	Dislocation and sprain of joints and ligaments of knee
S86	Injury of muscle, fascia, and tendon at lower leg level
S93	Dislocation and sprain of joints and ligaments at ankle, foot, and toe level

### Data analyses

For questions with categorical answers, data are presented as percentage of the total participant pool, or a portion of this pool. For questions with quantitative answers, data are presented as means with standard deviations. Sample sizes vary for different analyses due to the voluntary nature of the survey, but are indicated. All statistical analyses were performed using the JMP software program (JMP^®^, Version 13.1; SAS Institute Inc., Cary, NC, USA).

For our analyses, we categorized students by gender and by Beighton score. The Beighton score is the number of hypermobile joints out of the nine joints tested. It can range from 0 (no hypermobile joint) to 9 (all tested joints are hypermobile). Instead of subdividing respondents into two groups, i.e., participants with GJH (Beighton score ≥5) and participants without GJH (Beighton score 0–4), we present the data in three groups: (1) participants with a Beighton score of 0 (no joint laxity); (2) participants with a Beighton score of 1–4 (LJH); and (3) participants with a Beighton score of ≥5 (GJH).

Two-tail Fisher’s exact tests were used to examine whether the proportion of men and women reporting the presence and absence of different hypermobile joints differed. Pearson Chi-square tests were used to determine: (1) whether female and male participants differed in the proportion of respondents across the three Beighton score categories; (2) whether Beighton scores differed by ethnicity/race; (3) whether female and male participants differed in the rates of self-reported injury and types of injuries reported; (4) whether injury rates differed across the three Beighton score categories; (5) whether rates of athletic activity (categorical variable of whether or not a respondent was athletically active or not) differed between female and male participants and by Beighton score category; (6) whether rates of athletic activity differed with rates of musculoskeletal injury; (7) whether, among those who were athletically active, injury rates differed across the different categories of Beighton scores; and (8) rates of chronic joint and neck/back pain differences across the Beighton score categories. Odds ratios were calculated to measure the relative odds of the occurrence of hypermobility in nine joints given the gender of participants (i.e., females to males). The closer the value to 1, the lower the risk factor of a specific gender to the occurrence of hypermobility in a specific joint. Given the number of statistical analyses performed, we have applied a Bonferroni correction and use a *p* < 0.0015 as the threshold for significance.

## Results

### Epidemiologic data

Of the 686 students who participated in the survey, 32 respondents were excluded from the analyzed data set because they failed to provide an age, were younger than 18 years of age or were 26 years or older. Thus the analyzed pool of respondents consisted of 654 respondents, including 482 female (73.7% of respondent pool) and 172 male (26.3% of respondent pool) participants (Table 3). The mean age of respondents was 19.31 ± 1.24 years (mean ± standard deviation; range: 18–25 years; median age = 19 years). Most respondents were right-handed (*n* = 602 people, 92% of respondents), some were left-handed (*n* = 48, 7.3% of respondents), and a small number were ambidextrous (*n* = 4, 0.6%). The percentage of right-footed respondents was 84.4% (*n* = 552), 12.3% (*n* = 81) were left-footed and 1.7% (*n* = 11) were ambidextrous with their feet. Some respondents (*n* = 10, 1.5%) did not provide information on their footedness. The majority of respondents identified as Caucasian/White (*n* = 416, 63.6%), Hispanic (*n* = 88, 13.5%), African-American/Black (*n* = 58, 8.9%), or Asian (*n* = 14, 2.1%). All other respondents identified as more than one ethnicity/race or as an ethnicity/race other than the ones listed above (*n* = 78, 11.9%).

**Table 3:** Demographic data of survey respondents (*n* = 654).

**Gender:** Female 482 (73.7%), male 172 (26.3%) **Age:** 19.31 ± 1.24 years (mean ± standard deviation; range: 18–25 years; median age = 19 years) **Handedness:** Right-handed 602 (92%), left-handed 48 (7.3%), ambidextrous 4 (0.6%) **Footedness:** Right-footed 552 (84.4%), left-footed 81 (12.3%), ambidextrous 11 (1.7%), no data 10 (1.5%) **Ethnicity/race:** Caucasian/White 416 (63.6%), Hispanic 88 (13.5%), African-American/Black 58 (8.9%), Asian 14 (2.1%), Other/more than one race/ethnicity 78 (11.9%)

### Hypermobile joints

Almost half of the study participants (44.5%) were able to rest the palms of their hands flat on the floor with both knees fully extended, i.e., had a positive trunk flexion test (Table 4). The proportion of men and women with a positive trunk flexion test (25.1% in men vs. 52.5% in women) was statistically significant. Though female participants had greater rates of hypermobility of the knees, left elbow, little fingers, and thumbs than rates for male participants, the differences between the genders were not statistically significant for those joints (Table 4).

**Table 4:** Percentage of positive flexibility tests for each tested joint for all participants and male and female participant.

Joint	All participants		Male participants		Female participants		Statistical difference by gender*	Odds ratio (females to males)
	*n*	%	*n*	%	*n*	%		
Spine	647	44.5	171	25.1	476	51.5	***p* < 0.0001**	0.317
Right knee	648	18.8	170	10.6	478	21.7	***p* = 0.0013**	0.426
Left knee	647	19.2	170	12.4	477	21.6	*p* = 0.0089	0.512
Right little finger	653	22.8	172	19.8	481	24.0	*p* = 0.2909	0.784
Left little finger	651	22.6	172	18.0	479	24.2	*p* = 0.1107	0.688
Right thumb	653	20.7	172	19.2	481	21.2	*p* = 0.6609	0.882
Left thumb	652	22.7	172	18.0	480	24.4	*p* = 0.0908	0.682
Right elbow	651	16.0	172	8.1	479	18.8	***p* = 0.0010**	0.383
Left elbow	650	16.2	172	8.7	478	18.8	*p* = 0.0016	0.412

**Note:**

* Categorical Fisher exact tests (two tail) comparing the proportion of men and women with specific hypermobile joints. Total sample sizes provided in “All participants” column. A significance level of *p* < 0.0015 has been used. Bold *p* values indicate statistical significance.

### Beighton scores

**Figure 1:**
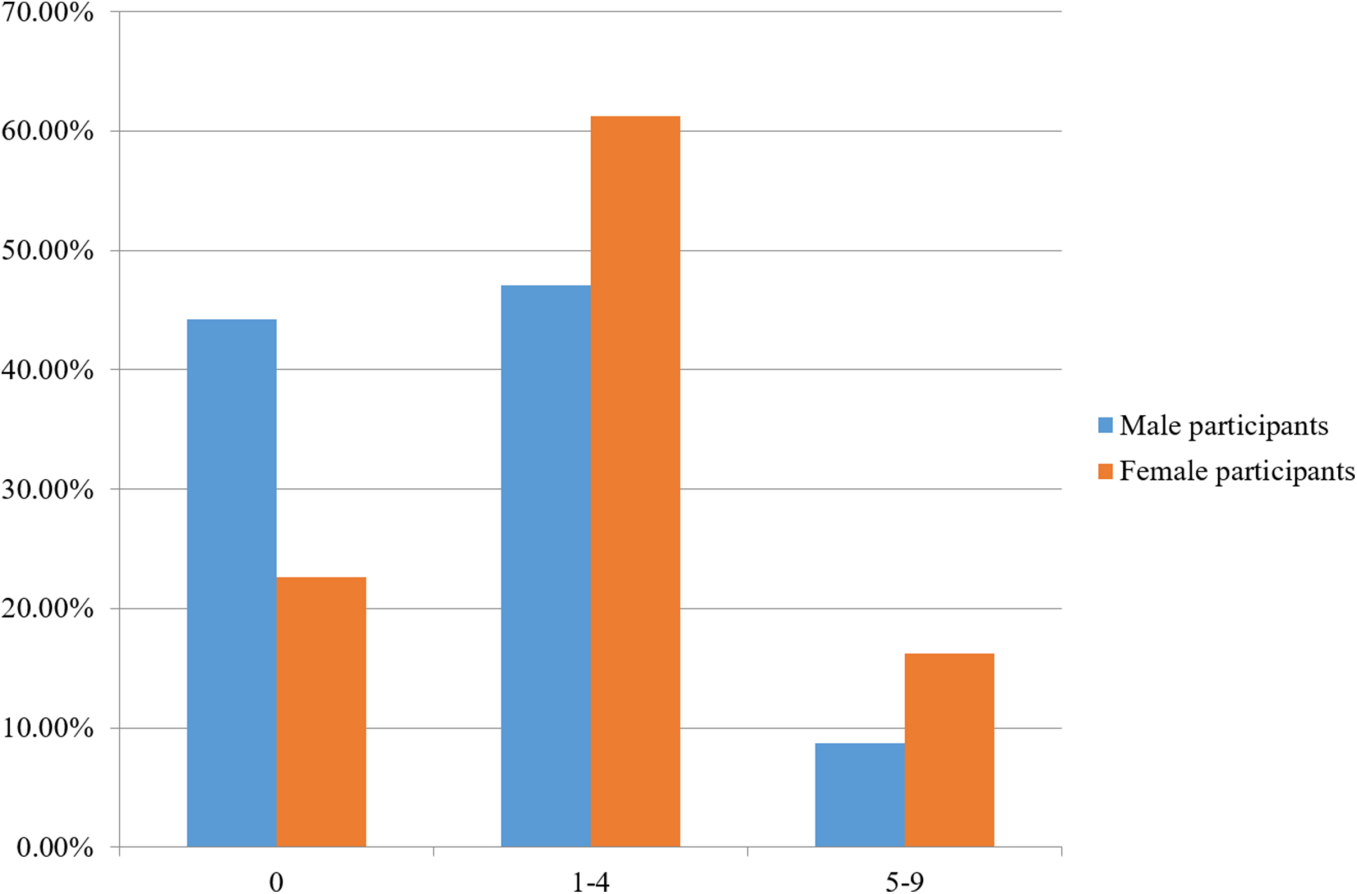
Percentage of male (*n* = 172) and female participants (*n* = 482) having a Beighton score of 0, 1–4, and 5–9.

Out of 654 participants, 28.3% had a Beighton score of 0 (no joint laxity), 57.5% had LJH (Beighton score of 1–4), and 14.2% of participants had a Beighton score of 5–9 (GJH). Female and male participants differed in the proportion of respondents that had Beighton scores of 0, 1–4, and 5–9 (Pearson Chi-square test, DF = 2, Chi-square = 30.207, *p* < 0.0001; Fig. 1).

Women did not have significantly higher rates of GJH (16.2%) than men (8.7%) (Pearson Chi-square test, DF = 1, Chi-square = 5.786, *p* = 0.0162). They did, however, have significantly higher rates of LJH (61.2% of women vs. 57.5% of men; Pearson Chi-square test, DF = 1, Chi-square = 10.327, *p* = 0.0013), and had a lower proportion of participants reporting a Beighton score of 0, compared to men (22.6% vs. 44.2%, respectively; Pearson Chi-square test, DF = 1, Chi-square = 29.080, *p* < 0.0001).

The Beighton scores (categorized as 0; 1–4; and 5–9) did not differ by ethnicity/race with respondents classified as African-American/Black, Asian, Caucasian/White, Hispanic, and Other/more than one race/ethnicity (Pearson Chi-square test, Chi-square = 13.015, *p* = 0.1113; Table 5).

**Table 5:** Beighton scores by race/ethnicity (number of respondents with percent of race/ethnicity in parentheses).

Race/ethnicity	0	1–4	5–9	Total
Asian	5 (35.7%)	6 (42.9%)	3 (21.4%)	14
African-American/Black	11 (19.0%)	34 (58.6%)	13 (22.4%)	58
Other/more than one race/ethnicity	26 (33.3%)	39 (50.0%)	13 (16.7%)	78
Hispanic	24 (27.3%)	48 (54.5%)	16 (18.2%)	88
Caucasian/White	119 (28.6%)	249 (59.9%)	48 (11.5%)	416
Total	185	376	93	654

There was no difference in Beighton scores when respondents in the “Other/more than one race/ethnicity” category were removed from analyses (Pearson Chi-square test, Chi-square = 10.916, *p* = 0.0910, *n* = 576).

### Beighton score and self-reported musculoskeletal injuries

#### Rates of self-reported injury

**Figure 2:**
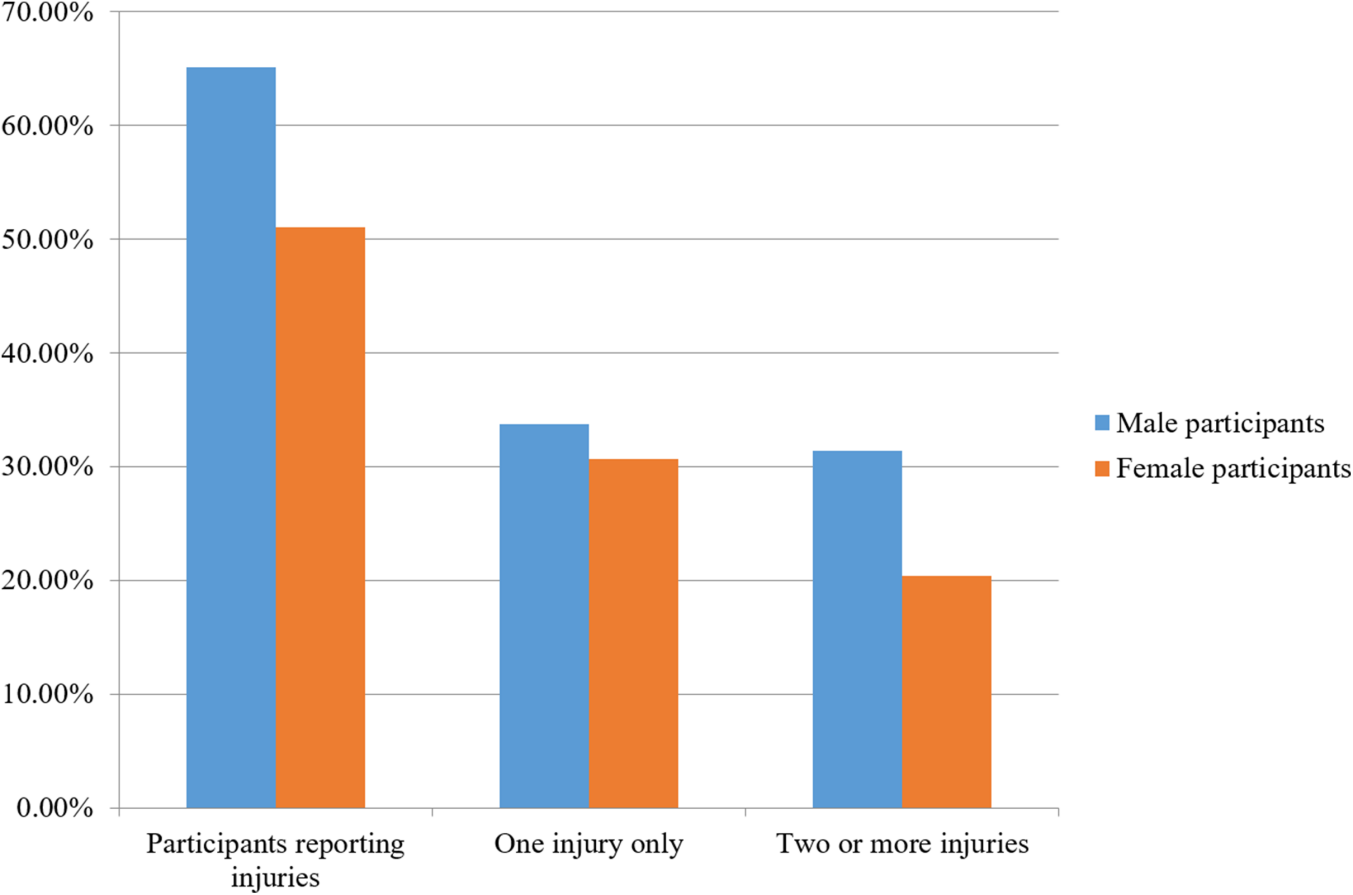
Percent of self-reported musculoskeletal injuries for male and female participants.

Just over half (54.7%, *n* = 354 participants) of the 654 participants indicated that they had suffered a musculoskeletal injury in the past in response to one or more of the following questions “Have you ever suffered a joint injury, such as a dislocation or fracture?,” “Have you ever suffered a ligament or tendon injury or inflammation, such as a sprain or tendon rupture?” and “Have you ever suffered a muscle injury, such as a pulled groin?” (see Appendix S1 for the complete survey). Most of these individuals (*n* = 206 participants) had been injured only once, though many (*n* = 152 participants) had been injured two or more times (Fig. 2).

Female participants reported lower rates of self-reported injuries (51.2%, *n* = 247 out of 482) than male respondents (64.5%, *n* = 111 out of 172; Pearson’s Chi-square test, DF = 1, Chi-square = 9.037, *p* = 0.0026; odds ratio: 1.73), although this difference was not significant.

#### Types of injury

Study participants who reported having been injured (*n* = 354 participants) reported a total of 611 different injuries. A total of 24 survey entries did not contain enough information to assign one of the 18 ICD-10-CM codes listed in Table 2.

The most commonly reported injury type for both women and men were S76 injuries, which include quadriceps, groin, and hamstring injuries (Table 6). The only other injury codes with an overall prevalence of ≥4% in our study population were S43 with 6.9%, S63 with 5.2%, S62 with 4.7%, and S86 with 4%. Altogether, 371 of 587 coded injuries (63.2%) belonged to one of three codes: S76 (injury of muscle, fascia and tendon at hip and thigh level), S93 (dislocation and sprain of joints and ligaments at ankle, foot, and toe level), and S83 (dislocation and sprain of joints and ligaments of the knee). Men and women did not differ in the types of injuries that they reported most (Pearson Chi-square test; DF = 10, Chi-square = 24.806, *p* = 0.0057; injuries categories grouped to include the top ten injuries reported by all respondents with all other injuries (S52, S29, S46, S16, S73, S03, S42, and S56) grouped into an “other” category; Table 6).

**Table 6:** Self-reported musculoskeletal injuries coded using ICD-10-CM codes for all injuries reported by participants, by gender, sorted by prevalence reporting from high to low.

ICD-10-CM code	Injuries reported by all participants (*n* = 587 injuries) (%)	Injuries reported by female participants (*n* = 402 injuries) (%)	Injuries reported by male participants (*n* = 185 injuries) (%)
S76	24.9	23.4	28.1
S93	19.3	21.6	14.1
S83	17.1	19.7	17.8
S43	7.7	4.7	14.1
S63	5.8	5.7	6.0
S62	5.3	5.0	6.0
S86	4.4	4.2	4.9
S82	3.7	4.7	1.6
S53	2.4	2.5	2.2
S39	2.0	2.2	1.6
S52	1.7	2.0	1.1
S29	1.4	1.2	1.6
S46	0.7	0.8	0.5
S16	0.5	0.5	0.5
S73	0.5	0.8	–
S03	0.3	0.5	–
S42	0.2	0.3	–
S56	0.2	0.3	–

#### Beighton score and self-reported injuries

There was no difference in the proportion of respondents classified within different categories of Beighton scores (categories: 0; 1–4; 5–9) and whether or not they reported having been injured (Pearson Chi-square test, DF = 2, Chi-square = 7.453, *p* = 0.0241).

**Figure 3:**
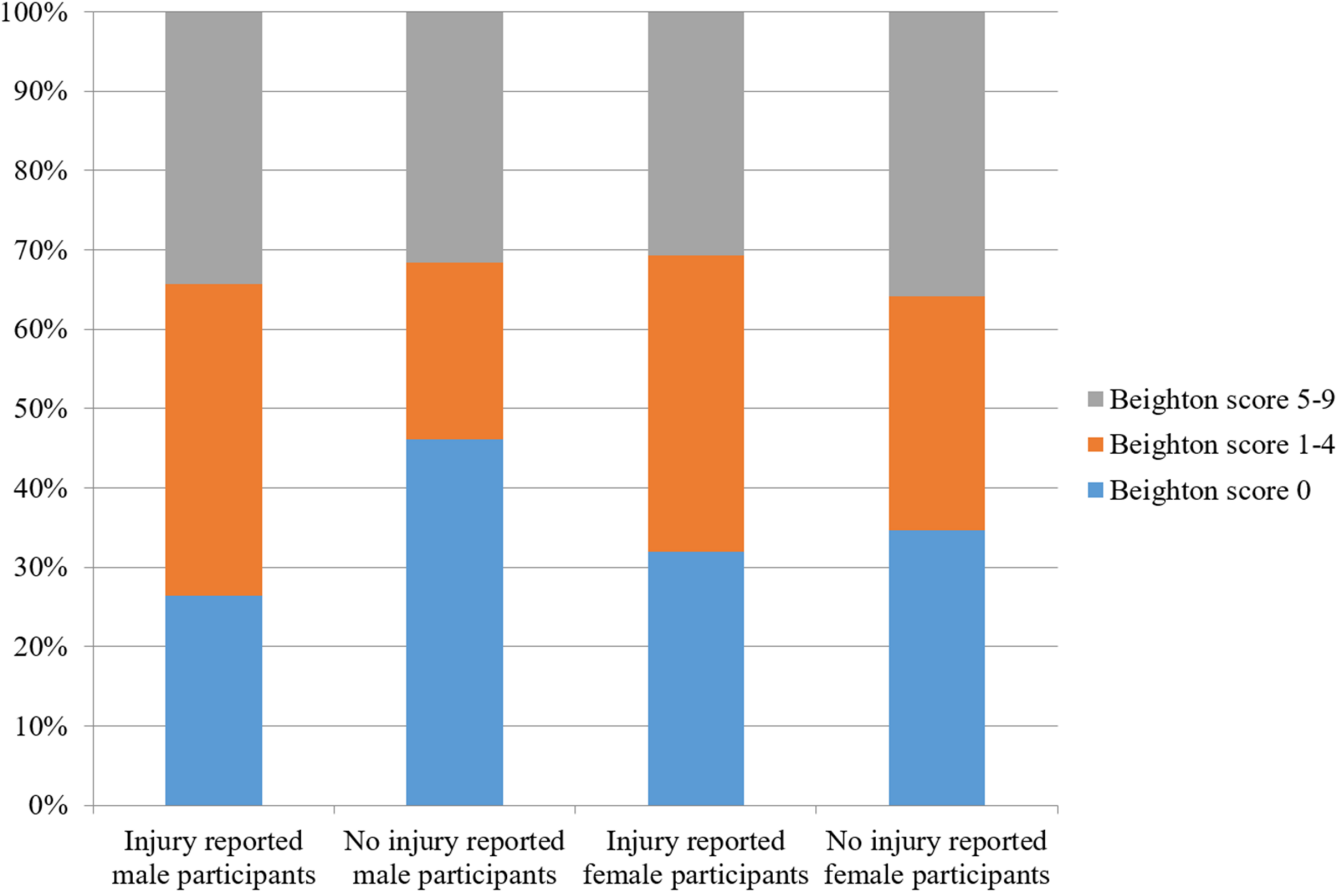
The proportion of male and female participants reporting injury or not reporting injury by Beighton score category.

Likewise, when examining women and men separately, the proportion of respondents reporting injuries also did not differ by their Beighton score category (Females: Pearson Chi-square test, DF = 2, Chi-square = 3.445, *p* = 0.1787; Males: Pearson Chi-square test, DF = 2, Chi-square = 10.936, *p* = 0.0042; Fig. 3).

When examining the three most commonly-reported injuries (S76, S93, and S83) within the list of injuries reported by participants, the proportion of injuries reported by participants that had Beighton scores of 0, 1–4, and 5–9 did not differ for S76 injuries (25.3%, 24.3%, and 20.7%, respectively; Pearson Chi-square test, DF = 2, Chi-square = 0.592, *p* = 0.7438); for S93 injuries (16.1%, 19.5%, and 18.4%, respectively; Pearson Chi-square test, DF = 2, Chi-square = 0.799, *p* = 0.6707); and for S83 injuries (16.11%, 18.4%, and 21.8%, respectively; Pearson Chi-square test, DF = 2, Chi-square = 1.209, *p* = 0.5465).

### Beighton score and chronic musculoskeletal pain

#### Chronic joint pain

Only 94 of 650 participants who responded to the questions “Do you suffer from chronic joint pain?” chose “yes” (14.5%; Table 7). The most commonly named joints were knee (*n* = 59), shoulder (*n* = 17), hip (*n* = 14), ankle (*n* = 9), and elbow (*n* = 7). Other entries named were wrist (*n* = 5), hand joints (*n* = 4), sacroiliac joint (*n* = 2), acromioclavicular joint (*n* = 1), and temporomandibular joint (*n* = 1).

**Table 7:** Proportion of all participants reporting the presence of chronic joint pain and chronic neck or back pain by Beighton score categories and by gender.

Beighton score	Chronic joint pain			Chronic neck/back pain		
	All (*n* = 650) (%)	Male (*n* = 172) (%)	Female (*n* = 478) (%)	All (*n* = 650) (%)	Male (*n* = 171) (%)	Female (*n* = 479) (%)
0	11.9	10.5	12.8	16.3	13.3	18.4
1–4	16.1	14.8	16.5	22.5	17.3	24.0
5–9	12.9	13.3	12.8	22.6	20.0	23.1
All	14.5	12.8	15.0	20.8	15.8	22.5

**Figure 4:**
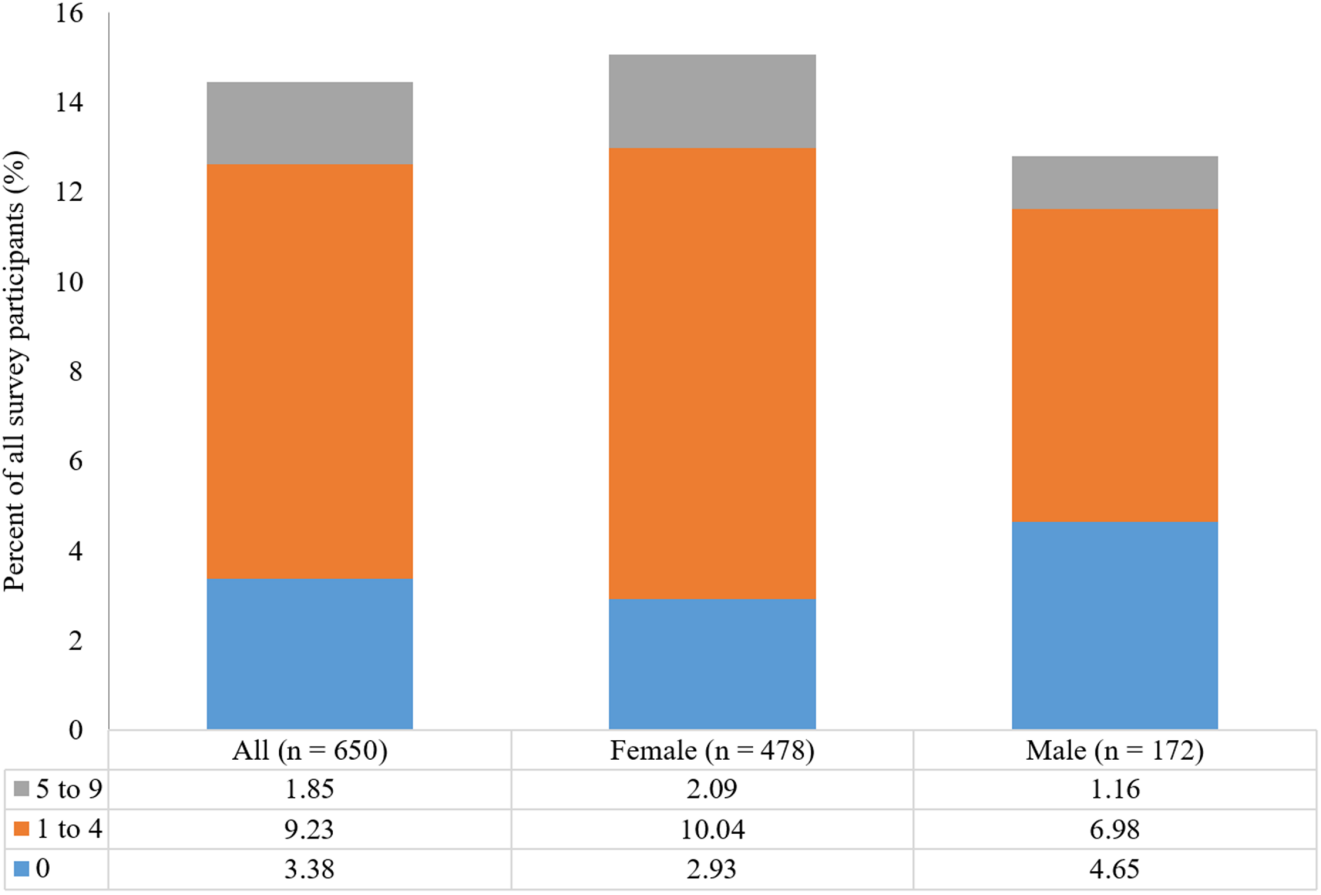
Proportion of all participants reporting the presence of chronic joint pain by Beighton score categories and by gender.

There was no difference in the proportion of respondents who reported chronic joint pain across the three Beighton score categories (11.9%, 16.1%, and 12.9% of respondents with Beighton scores of 0, 1–4, and 5–9, respectively; Pearson Chi-square test, DF = 2, Chi-square = 2.006, *p* = 0.3667; Table 7). There was also no difference in the proportion of respondents reporting chronic joint pain injury across the three Beighton score categories when female and male participants were analyzed separately (Females: Pearson Chi-square test, Chi-square = 1.192, *p* = 0.5509, *n* = 478; Males: Pearson Chi-square test, Chi-square = 0.651, *p* = 0.7222, *n* = 172; Fig. 4).

#### Chronic neck and back pain

The overall prevalence of chronic neck or back pain in our study population was 20.8% (135 of 650 respondents; Table 7). Pain in the lower back/lumbar area was reported by 79 respondents, pain in the neck by 33 respondents, and pain in the upper back/thoracic area by 15 respondents. A total of 37 respondents described chronic pain in the back without narrowing it down to a specific region.

**Figure 5:**
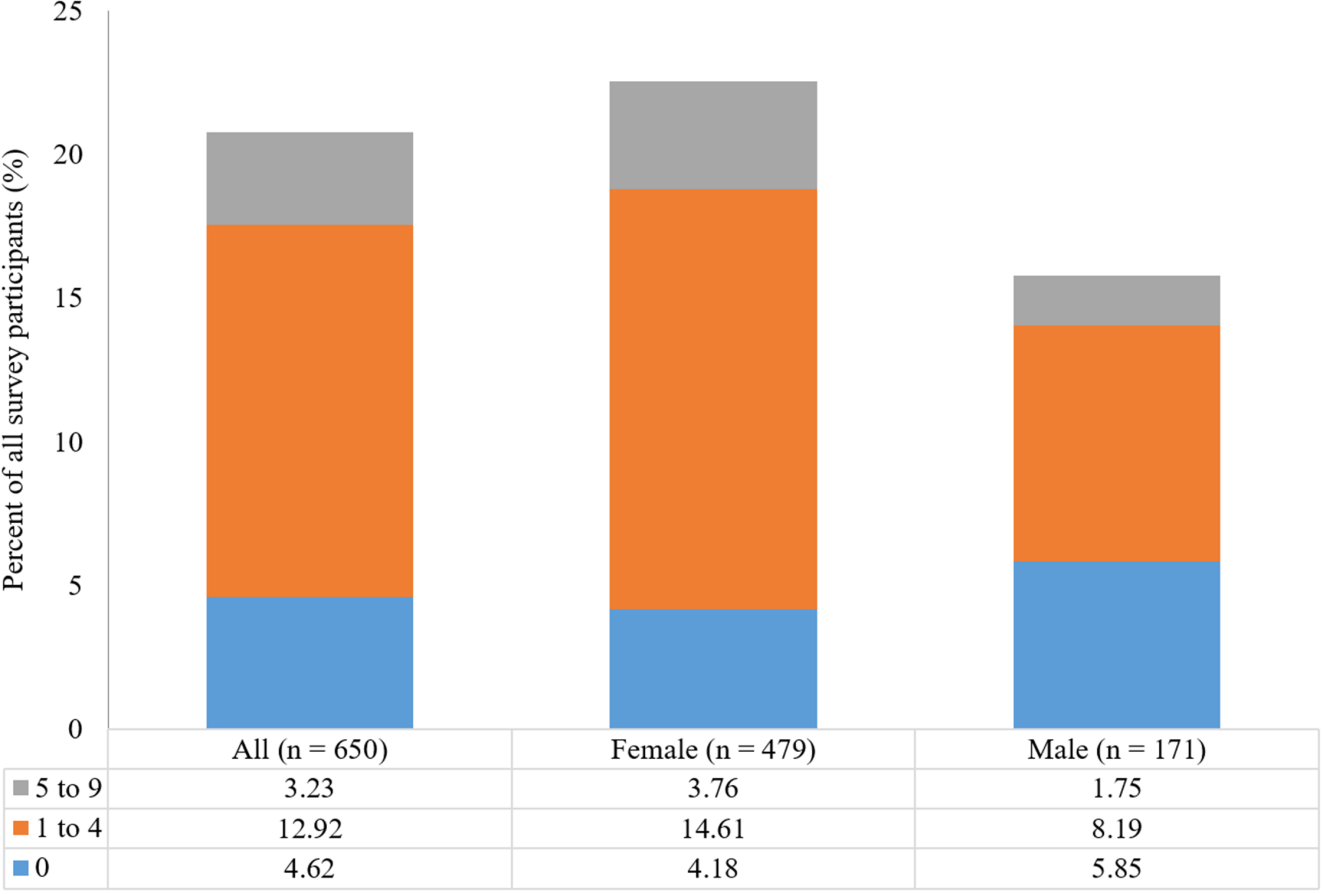
Proportion of all participants reporting the presence of chronic neck and back pain by Beighton score categories and by gender.

There was no difference in the proportion of respondents who reported chronic neck and back pain across the three Beighton score categories (16.3%, 22.5%, and 22.6% of respondents with Beighton scores of 0, 1–4, and 5–9, respectively; Pearson Chi-square test, DF = 2, Chi-square = 3.109, *p* = 0.2113; Table 7). There was also no difference when examining female and male participants separately (Females: Pearson Chi-square test, Chi-square = 1.453, *p* = 0.4837, *n* = 479; Males: Pearson Chi-square test, Chi-square = 0.676, *p* = 0.7131, *n* = 171; Fig. 5).

#### Pain intensity

Participants were also asked to enter information about the intensity of chronic pain on a scale from 0 to 10 (see Appendix S1 for the complete survey). Not all participants who indicated suffering from chronic joint or chronic neck or back pain provided information on pain intensity. Male (*n* = 24) and female (*n* = 99) participants did not differ in their reported chronic joint pain intensity (Wilcoxon rank sums test, DF = 1, Chi-square = 0.4685, *p* = 0.4968; Table 8). However, they did differ in their reported chronic neck and back pain intensity (Wilcoxon rank sums test, DF = 1, Chi-square = 7.3980, *p* = 0.0066) with male participants reporting lower (3.7 ± 1.8) pain intensity than female participants (4.6 ± 1.7).

**Table 8:** Beighton scores and self-reported pain intensity for chronic joint pain (*n* = 92) and chronic neck or back pain (*n* = 113) for all respondents.

Beighton score	Chronic joint pain		Chronic neck/back pain	
	*n*	Average pain intensity	*n*	Average pain intensity
0	21	4.5 ± 1.8 (2–8)	28	4.2 ± 1.7 (1–8)
1–4	59	4.3 ± 1.6 (2–8)	70	4.5 ± 1.7 (2–10)
5–9	12	4.3 ± 1.8 (2–6)	20	4.8 ± 2.2 (2–9)
All	92	4.4 ± 1.6 (2–8)	123	4.5 ± 1.8 (1–10)

**Note:**

Table depicts means ± st. dev with the range of pain values reported by respondents in parentheses.

The average chronic joint pain intensity did not differ across students with a Beighton score of 0, 1–4, and 5–9 (Kruskal–Wallis rank sums test, DF = 2, Chi-square = 0.0922, *p* = 0.9550; Table 8). The average neck and back pain intensity did not differ across students with a Beighton score of 0, 1–4, and 5–9 (Kruskal–Wallis rank sums test, DF = 2, Chi-square = 0.2453, *p* = 0.8846, and there was no effect once adjusting on gender (standard least squares test, DF = 2, *F*-ratio = 0.3350, *p* = 0.7160, gender as a random effect).

## Discussion

This study increases knowledge about a correlation between GJH, musculoskeletal injuries, and chronic pain of joints, back, and neck, on the strength of data collected from 654 undergraduate students at an American university. In our study we find that female participants had higher rates of hypermobility of joints of the spine and right elbow. One seventh (14.2%) of participants showed GJH based on a cut-off Beighton score of ≥5 with a lower proportion of female (22.6%) than male participants (44.2%) reporting no hypermobile joints (Beighton score of 0). The Beighton scores did not differ by ethnicity/race, though. There was no difference in the proportion of respondents classified within different categories of Beighton scores (categories: 0; 1–4; 5–9) and reported rates of injury. Finally, there was no difference in the proportion of respondents who reported having chronic pain of joints and neck or back across the three Beighton score categories. In addition to discussing the implications of our findings, we note some of the benefits of using a structured activity (such as the one used in this study) to collect participant data.

### Epidemiologic data

The composition of our study population, with 73.7% female and 26.3% male participants, does not reflect the demographics of the FGCU student body (53% female students and 47% male students) or the United States population (50.8% females, 49.2% males) (Florida Gulf Coast University, 2018; United States Census Bureau, 2018). The main reason for having a higher percentage of female participants is that the participants of our study were predominantly pre-health professions majors (particularly pre-nursing students), which is a field that continues to have an above-average proportion of female employees (Rappleye, 2015).

The demographics for ethnicity/race in our study were close to those of the overall FGCU student population (White 63.2%, Hispanic 21%, Black 8.5%, Asian 2.9%, non-resident alien 2%, not reported 1.4%, Native American 1%) and of the population of the United States (White 60.7%, Hispanic 18.1%, Black/African-American 13.4%, Asian 5.8%, Other/more than on race/ethnicity 4.2%) (Florida Gulf Coast University, 2018; United States Census Bureau, 2018).

### Hypermobile joints

Despite the condition being termed “GJH,” it is more often pauciarticular than polyarticular, and not all joints used as part of the Beighton criterion are equally affected (Russek & Errico, 2016; Al-Rawi, Al-Aszawi & Al-Chalabi, 1985; Cameron et al., 2010; Quresh, Maalik & Ahmad, 2010). Published studies, however, differ considerably on the joints that have a higher percentage of hypermobility; these differences are often due to the age of the study population and the race/ethnicity of study participants. The only recent study (2016) with a study population similar to that of our study (i.e., mainly Caucasian/white undergraduate and graduate students at an American university) reported hypermobility rates of 24.4% for the elbow joint (vs. 16.1% for our study), 24.7% for the knee joint (vs. 19.0% for our study), 28.5% for the spine (vs. 44.5% for our study), 53.2% for the little finger (vs. 22.7% for our study), and 50.2% for the thumb (vs. 21.7% for our study) (Russek & Errico, 2016). The study did not report separates rates for males and female participants, only for the total study population.

Assessment of joint mobility of participants in our study was done as a group assessment (two to four students working together) using goniometers under supervision by trained members of the research team. Using this procedure may account for the lower prevalence rates found compared to studies that relied upon self-assessment by participants, who may consider joints to be hypermobile even though they do not meet the Beighton criteria for hypermobility.

### Beighton scores

Before the International Consortium on the EDSs proposed cut-off Beighton scores for the diagnosis of GJH of ≥6 for pre-pubertal children and adolescents, ≥5 for pubertal men and women up to the age of 50, and ≥4 for those >50 years of age in 2017, studies used a variety of Beighton scores such as ≥3, ≥4 or ≥6 as cut-off scores. The current study used a Beighton score of ≥5; however, the discussion will use cut-off scores of ≥4 and ≥5 to improve the comparability of the results.

Because the female-to-male ratio in our study population was 74:26, the overall prevalence of GJH of 14.2% for a cut-off score of ≥5 and of 20.8% for a cut-off score of ≥4 in our sample needs to be adjusted to compensate for that ratio. Based on the GJH prevalence determined for male (8.7% for ≥5; 12.2% for ≥4) and female participants (16.2% for ≥5; 23.9% for ≥4) and a female-to-male ratio of 51:49 in the US population, our study allows us to estimate a prevalence of GJH in a university-aged US population as 12.5% using a Beighton score of ≥5 and as 18.2% using a Beighton score of ≥4 (United States Census Bureau, 2018). This percentage is at the lower end of the range of prevalence rates reported in other studies, although most of the studies looked at populations that were different from ours as far as age range and race/ethnicity is concerned (Scheper et al., 2014; Remvig et al., 2011; Rikken-Bultman, Wellink & Van Dongen, 1997; Simmonds & Keer, 2007; Lamari, Chueire & Cordeiro, 2005; Quresh, Maalik & Ahmad, 2010). Compared to the last two studies involving similar populations at North American universities, the prevalence found in our study is considerably higher than the rate reported in a study involving freshmen at a military academy (1.5%) but lower than the rate reported for a study involving healthy undergraduate and graduate students at a private university (26.2% using a cut-off Beighton score of ≥5 and 40.1% for a cut-off score of ≥4) (Russek & Errico, 2016; Cameron et al., 2010). The participants of the first study cannot be considered to be representative of the general university population in the United States due to the requirements for physical aptitude for applicants (United States Military Academy at West Point, 2018). For example, and for comparison, Larsson, Baum & Mudholkar (1987) reported a prevalence of 19.1% for 660 US music students ages 14–68 using a Beighton score of ≥3. A study by Decoster et al. (1997) involved 264 US adolescent athletes with a mean age of 15.5 years; using a cut-off score of ≥5 it found a GJH prevalence of 12.9%. A 1999 study of 310 male and female NCAA lacrosse players determined an overall GJH prevalence of 23.8% using a cut-off score of ≥5 (Decoster et al., 1999).

Female participants in our study did not have significant higher rates of GJH (16.2% for ≥5; 23.9% for ≥4) than men (8.7% for ≥5; 12.2% for ≥4). Previous studies reported similar difference between women and men (Beighton, Solomon & Soskolne, 1973; Jansson et al., 2004; Rikken-Bultman, Wellink & Van Dongen, 1997; Arroyo, Brewer & Giannini, 1988; Wordsworth et al., 1987; Jessee, Owen & Sagar, 1980; Al-Rawi, Al-Aszawi & Al-Chalabi, 1985; Larsson, Baum & Mudholkar, 1987; Gedalia et al., 1985; Decoster et al., 1997; Hakim, Malfait & Paepe, 2010; Didia, Dapper & Boboye, 2002). For example, Russek & Errico (2016) found prevalence rates of 36.7% for females and 13.7% for males using a Beighton score of ≥5. Although those rates are considerably higher than ours, the ratio of female-to-male prevalence is similar to the one in our study (1.9:1 vs. 2:7). Larsson, Baum & Mudholkar (1987) reported a female:male Beighton score ratio of 3.6:1 for US music students (ages 14–68), Decoster et al. (1997) found a female:male Beighton score ratio of 3.7:1 among US adolescent athletes, and Didia, Dapper & Boboye (2002) reported a female:male Beighton score ratio of 2.1:1 among undergraduate students in Nigeria.

Although participants identifying as Caucasian/White did have lower rates of GJH (11.5%) than participants identifying as African-American/Black (22.4%), Asian (21.4%), and Hispanic (18.2%), these differences were not statistically significant, in contrast with previous studies that reported significantly higher rates of GJH for Black/African and Asian populations (Russek, 1999; Wordsworth et al., 1987; Hakim, Malfait & Paepe, 2010; Grahame, 1990). Future studies with a larger sample size and more diverse participants pool, might be able to better address this issue.

### Beighton score and self-reported musculoskeletal injuries

Although there are studies suggesting an increased injury risk for people with GJH, especially for sports-related injuries to the ankle (sprains, fractures), knee (ACL injury), and shoulder joint (rotator cuff injury, dislocation), there are also studies that show a similar or reduced injury risk for athletes with GJH (Scheper et al., 2013; Decoster et al., 1999; Smith et al., 2005; Beynnon, Murphy & Alosa, 2002; Borsa, Sauers & Herling, 2000; Wolf, Cameron & Owens, 2011; Konopinski, Jones & Johnson, 2012; Pacey et al., 2010; Ramesh et al., 2005; Myer et al., 2008; Nicholas, 1970; Stewart & Burden, 2004; Krivickas & Feinberg, 1996). The finding of our study that male participants with a Beighton score of 1–9 are significantly more likely to having suffered a musculoskeletal injury than male participants with a Beighton score of 0 is similar to the results of a study involving 52 rugby players in England although the study populations are different (Stewart & Burden, 2004). In contrast, a study comparing the injury rates of professional football players in England found almost identical injury rates for players with and without hypermobile joints (Collinge & Simmonds, 2009).

The results of our study, i.e., that young female adults with GJH do not have an increased risk for musculoskeletal injuries, are similar to results reported in studies involving dance students (Ruemper & Watkins, 2012), NCAA lacrosse players (Decoster et al., 1999), and elite female soccer players in Holland (Blokland et al., 2018).

Our study did not confirm reports that people with GJH are more likely to sustain ankle injuries such as sprains (Decoster et al., 1999; Azma et al., 2014). Study participants with a Beighton score of 1–4 had the highest prevalence for all three major injury groups overall and for both genders. Male participants of this group reported higher rates for S76 injuries of muscles, fascia and tendon at the hip and thigh level, while female participants recalled slightly more S93 (dislocation and sprain of joints and ligaments at ankle, foot, and toe level) and S83 (dislocation and sprain of joints and ligaments of the knee) injuries. None of these differences were significant, however.

### Beighton score and chronic musculoskeletal pain

There is a plethora of studies reporting that patients with BJHS suffer from chronic MSP that may affect their daily lives (Kumar & Lenert, 2017; Morris et al., 2017; Leone et al., 2009; Hakim, Malfait & Paepe, 2010; Grahame & Jenkins, 1972; Scheper et al., 2013; Chustecka, 2004; Grahame, 2000b; Simonsen et al., 2012; Nikolajsen et al., 2013; Voermans et al., 2010; Engelbert et al., 2003; Hanewinkel-Van Kleef et al., 2009). On the other hand, studies looking at a correlation of GJH and MSP have shown inconclusive results only. For example, a systematic review did not show an association in Caucasian children and only a potential association in African and Asian children (McCluskey et al., 2012). A 4-year longitudinal study of schoolchildren in Finland indicated that having GJH was a predictor of MSP (El-Metwally et al., 2004). GJH was found to be a risk factor for MSP during adolescence in children from the Avon Longitudinal Study of Parents and Children (Tobias et al., 2013). Another longitudinal study involving preadolescents in Denmark failed to show a statistically significant association between GJH and arthralgia for that age group (Sohrbeck-Nohr et al., 2014). The finding of our study that there is no correlation between GJH and MSP in a university-aged population lines up with the results of those studies. However, other studies did not report a higher pain intensity for female participants for chronic neck and back pain like we found in our study.

### Study limitations and the utility of the Beighton score

The three main limitations of our study are (1) participant selection, (2) reliance on recall of injuries, and (3) reliance on self-reported chronic pain. First, asking students in an undergraduate university course that is a prerequisite requirement for health professions majors to participate in a study narrows down the age range of participants and excludes students from other areas of study. The mean age of participants (19.31 ± 1.24 years) is close to the upper range of adolescence and our result can only be considered to be applicable to young adults. Second, having participants recount past injuries inadvertently introduces recall bias into our study. Some participants may not be aware of injuries sustained when they were younger or may recall injuries incorrectly. However, there is no indication that participants with different Beighton scores may have recalled past injuries to varying degrees. Third, even though our survey contains a definition of chronic pain as pain lasting three months or more, participants may have responded positively without having suffered from pain for such an extended period of time. Again, however, there is no indication that participants with different Beighton scores may be more or less prone to misjudge the length of time they have been suffering from pain.

Also, the scoring system currently in use to determine the Beighton score has been criticized for not including more joints in other parts of the body (Remvig et al., 2014). With GJH most often being pauciarticular, it is conceivable that limiting the number of joints assessed could increase the likelihood of false positive or false negative results. Historically, the lack of a defined cut-off Beighton score for the diagnosis of GJH was a point of great concern due to hypermobility being more prevalent in younger populations and showing a decrease in prevalence over time (Scheper et al., 2014; Remvig et al., 2011; Rikken-Bultman, Wellink & Van Dongen, 1997; Simmonds & Keer, 2007; Lamari, Chueire & Cordeiro, 2005; Leone et al., 2009). Using different cut-off cores created ambiguity in the literature. Going forward, researchers and practitioners should adhere to the criteria of the 2017 International Classification. They should not only indicate which Beighton cut-off score has been used in an assessment, but also clearly state the age ranges and study population upon which an assessment has been done. If possible, data should be presented by gender, given that numerous studies show females have higher Beighton scores than males.

## Conclusion

Our study determined the prevalence of GJH in a university-aged population in North America as 12.5% for the overall population and as 16.2% for women and 8.7% for men. The most common hypermobile joints are the joints of the spine, especially in women. Women also have a significantly higher rate of hypermobility of the right knee and elbow joints. Our study did not show a significant difference in the prevalence of hypermobility between African-American/Black, Asian, Caucasian/White, and Hispanic participants although Caucasian/White respondents had the lowest prevalence of GJH. This question could be explored further in future studies. Young male adults with GJH are at an increased risk for musculoskeletal injuries, whereas hypermobility in young female adults is not associated with an increased prevalence of musculoskeletal injuries. Young adults with GJH are not reporting higher rates of chronic pain in joints, the neck or the back regardless of their gender nor do they suffer from more severe pain.

## Supplemental Information

10.7717/peerj.7625/supp-1 Supplemental Information 1 Demographic data.

10.7717/peerj.7625/supp-2 Supplemental Information 2 Gender, ethnicity, and Beighton score.

10.7717/peerj.7625/supp-3 Supplemental Information 3 Gender, Beighton score, and injuries ICD-10 coded.

10.7717/peerj.7625/supp-4 Supplemental Information 4 Gender, Beighton score, and chronic pain.

10.7717/peerj.7625/supp-5 Supplemental Information 5 Gender, Beighton score, and pain intensity.

10.7717/peerj.7625/supp-6 Supplemental Information 6 Anthropometric and physiologic differences in men and women.

## Additional Information and Declarations

### Data Availability

The following information was supplied regarding data availability: The raw data are available in the Supplemental Tables.
