# Supplementary material for: Prevalence of generalized joint hypermobility, musculoskeletal injuries, and chronic musculoskeletal pain among American university students
Source: PeerJ. 2019 Sep 11;7:e7625. doi: 10.7717/peerj.7625 (PMC6744937; doi:10.7717/peerj.7625)
Supplement: Supplemental Information 6 [file peerj-07-7625-s006.pdf]

Anthropometric and Physiologic Differences in Men and Women

Please take your time to read the following information carefully before starting the survey.

- We are conducting an Undergraduate Research Project with the title “Anthropometric and Physiologic Differences in Men and Women” and would like to anonymously collect information and data from you. Although participants will not gain any direct benefits from participating in the study, society will benefit from the knowledge gained from this study as we aim to answer questions that have not been answered so far conclusively or have not been studied yet.

- Because you submit your answers anonymously, neither we nor anybody else who will look at individual surveys or the compiled results of all surveys will be able to identify you or any other participant in this study. To make sure that this survey truly is anonymous and that no participant can be identified by either us or anybody looking at the survey forms please:

  - DO NOT PUT YOUR NAME ON THE SURVEY.
  - DO NOT SIGN THE SURVEY WITH YOUR NAME.

- This survey has a very low likelihood and seriousness of probing for personal or sensitive information because it is completely anonymous, participation is optional, and participants have the freedom to control the level of disclosure (i.e., skip all or some questions). There may, however, be individuals who find the questions to be too personal. These individuals have the right to end participation without any repercussion and can deposit a blank survey form into the survey box.

- Your participation in the study is completely voluntary. If you decide to participate now you may change your mind and stop at any time, for any reason, without penalty or loss of any future services you may be eligible to receive from the University.

- There is no connection between your participation in the survey and the grade you will receive in your A&P class.

- You do not have to answer all of the questions in this survey. If you do not feel comfortable answering a specific question, please leave it blank. If you do not feel comfortable taking the survey entirely just deposit the blank survey into the survey box.

- Please make sure to deposit your copy of the survey regardless of whether you answered all, some or none of the questions into the survey box as this helps mask who did and who didn’t take the survey.

- If you have any questions about this study, you may contact the faculty sponsor Dr. Peter Reuter at 590-7512 or via email at preuter@fgcu.edu.

- If you have any questions about your rights as a subject/participant in this research, or if you feel you have been placed at risk, you can contact the Chair of the Human Subjects’ Institutional Review Board through Sandra Terranova, Office of Research and Sponsored Programs, at 239-590-7522.

- You may keep this form in case you have further questions and want to contact either Dr. Reuter or Sandy Terranova at the Office of Research and Sponsored Programs.

- Completing the survey should take no more than 15 minutes.

You must be of legal age to participate in this survey. By putting the survey into the survey box you confirm that you are 18 years of age or older, that you freely consent to participate in the study, and that you give us permission to use the data provided in publications and/or presentations.

1. **Ulnar collateral ligament no stress** (2 measurements)
   - **Right elbow** **length**: _________ mm _________ mm **thickness**: _________ mm _________ mm
   - **Left elbow** **length**: _________ mm _________ mm **thickness**: _________ mm _________ mm
2. **Ulnar collateral ligament valgus stress** (2 measurements)
   - **Right elbow** **length**: _________ mm _________ mm **thickness**: _________ mm _________ mm
   - **Left elbow** **length**: _________ mm _________ mm **thickness**: _________ mm _________ mm
3. **Hand Grip Strength** (2 measurements):
   - **Right hand** _________ lbs _________ lbs
   - **Left Hand** _________ lbs _________ lbs
4. **Can you place both palms flat on the floor with your legs straight while standing up?**
   - No: _______
   - Yes:_______
5. **Can you hyperextend your knee, i.e., bend it backwards more than 10°?**
   - **Right knee**: Yes: _______ No:_______
   - **Left knee**: Yes: _______ No:_______
6. **Can you bend your little finger backwards beyond 90 ^{o}?**
   - **Right hand**: Yes: _______ No:_______
   - **Left hand**: Yes: _______ No:_______
7. **Can you bend your thumb backwards so it touches the forearm?**
   - **Right thumb**: Yes: _______ No:_______
   - **Left thumb**: Yes: _______ No:_______
8. **Can you hyperextend your elbow, i.e., bend it backwards more than 10°?**
   - **Right elbow**: Yes: _______ No:_______
   - **Left elbow**: Yes: _______ No:_______

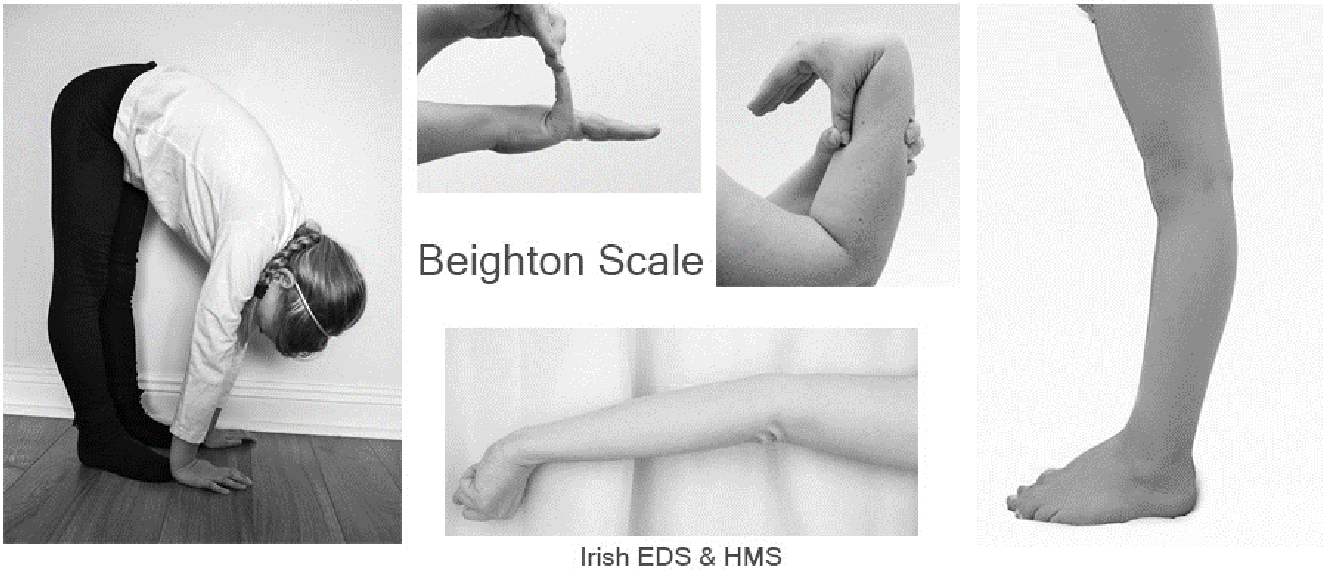

I. Demographic Information

1. **Gender**: Male___________ Female___________
2. **Weight**: ___________ lbs
3. **Height**: ___________ ft’ in”
4. **Age**: _________ yrs.
5. **Are you right or left handed?** _______ Right _______ Left
6. **Are you right or left footed?** _______ Right _______ Left
7. **Ethnicity/Race (mark all that apply)**
   - Caucasian/White
   - African-American/Black
   - Hispanic
   - Non-Hispanic
   - Other ____________________________________

II. Athletic Involvement

1. Are you actively involved in a team or individual athletic activity?
   - No: _______
   - Yes:_______
     - Which sport do you participate in? _______________________

       (If you participate in more than one sports write down the one you are most active in)

2. Which of the following applies to your team/sport?
   - Official NCAA FGCU Athletics Team _______
   - Registered FGCU Sports Club _______
   - Club/team outside FGCU _______

3. Which position do you play or event do you participate in? (Pitcher, point guard, 500m free-style, N/A, etc.)

   ____

4. How many hours per week do you spend practicing, endurance training/conditioning, and lifting weights/strength training?
   - Practice: _______ hours
   - Endurance/Conditioning: _______ hours
   - Strength/Lifting Weights: _______ hours

III. Injury History

1. Have you ever suffered a joint injury, such as a dislocation or fracture?
   - No: _______
   - Yes:_______
     - Which joint(s)?
     - What kind of injury?
     - At what age?

2. Have you ever suffered a ligament or tendon injury or inflammation, such as a sprain or tendon rupture?
   - No: _______
   - Yes:_______
     - Which ligament/tendon?
     - What kind of injury?
     - At what age?

3. Have you ever suffered a muscle injury, such as a pulled groin?
   - No: _______
   - Yes:_______
     - What kind of injury?
     - At what age?

4. Have you ever suffered from a hernia, such as inguinal hernia?
   - No: _______
   - Yes:_______
     - What kind of injury?
     - At what age?

5. Have you ever suffered from a herniated disc in your back or spine?
   - No: _______
   - Yes:_______
     - Where?
     - At what age?

IV. Medical History

1. Have you been diagnosed with a connective tissue disorder, such as Ehlers-Danlos Syndrome, Marfan Syndrome, etc.?
   - No: _______
   - Yes:_______
     - Which disorder?
     - At what age?

2. Does anyone in your family have hypermobile joints?

- No: _______
- Yes:_______
    - What is their relationship to you?
    - Which joint(s) are hypermobile?

3. Has anyone in your family undergone a joint replacement, such as hip or knee?

- No: _______
- Yes:_______
    - What is their relationship to you?
    - Which joint(s)?

4. Do you have to wear glasses/contact lenses?

- No: _______
- Yes, because I'm nearsighted:_______ (you can't see objects further away without glasses)
- Yes, because I'm farsighted:_______ (you can't see objects close to you without glasses)

5. Does anyone in your family have to wear glasses/contact lenses?

- No: _______
- Yes:_______
    - What is their relationship to you?

6. Has anyone in your family suffered from a hernia, such as inguinal hernia?

- No: _______
- Yes:_______
    - What is their relationship to you?

7. Has anyone in your family suffered from a herniated/prolapsed disc in their neck or back?

- No: _______
- Yes:_______
    - What is their relationship to you?

8. Do you have varicose veins?

- No: _______
- Yes:_______

9. Does anyone in your family have varicose veins?

- No: _______
- Yes:_______
- What is their relationship to you?

10. Do you suffer from chronic joint pain? (pain lasting 3 months or more)

- No: _______
- Yes:_______
  - Which joint(s)?
  - How bad is the pain most of the time using below pain scale?______

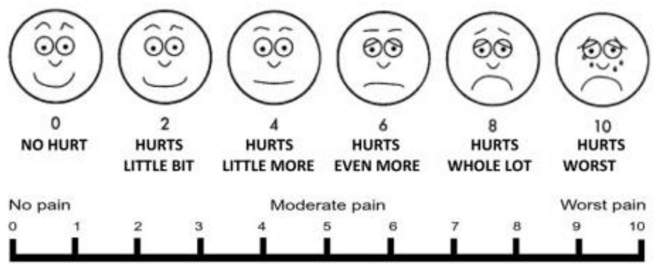

11. Do you suffer from chronic neck or back pain? (pain lasting 3 months or more)

- No: _______
- Yes:_______
  - Which area?
  - How bad is the pain most of the time using below pain scale?______

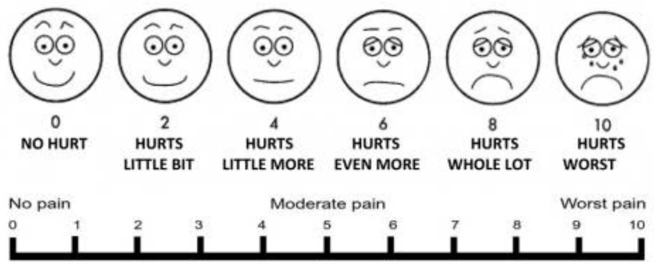

12. If you answered “yes” to questions 10 and/or 11 above, can you tell us if and how this chronic pain affects your daily life, studying, sleep etc.?
